# Supplementary material for: Optical imaging of single-protein size, charge, mobility, and binding
Source: Nat Commun. 2020 Sep 21;11:4768. doi: 10.1038/s41467-020-18547-w (PMC7505846; doi:10.1038/s41467-020-18547-w)
Supplement: Supplementary file 1 — Supplementary Information [file 41467_2020_18547_MOESM1_ESM.pdf]

Supplementary Information

Optical imaging of single protein size, charge, mobility and binding

Ma et al.

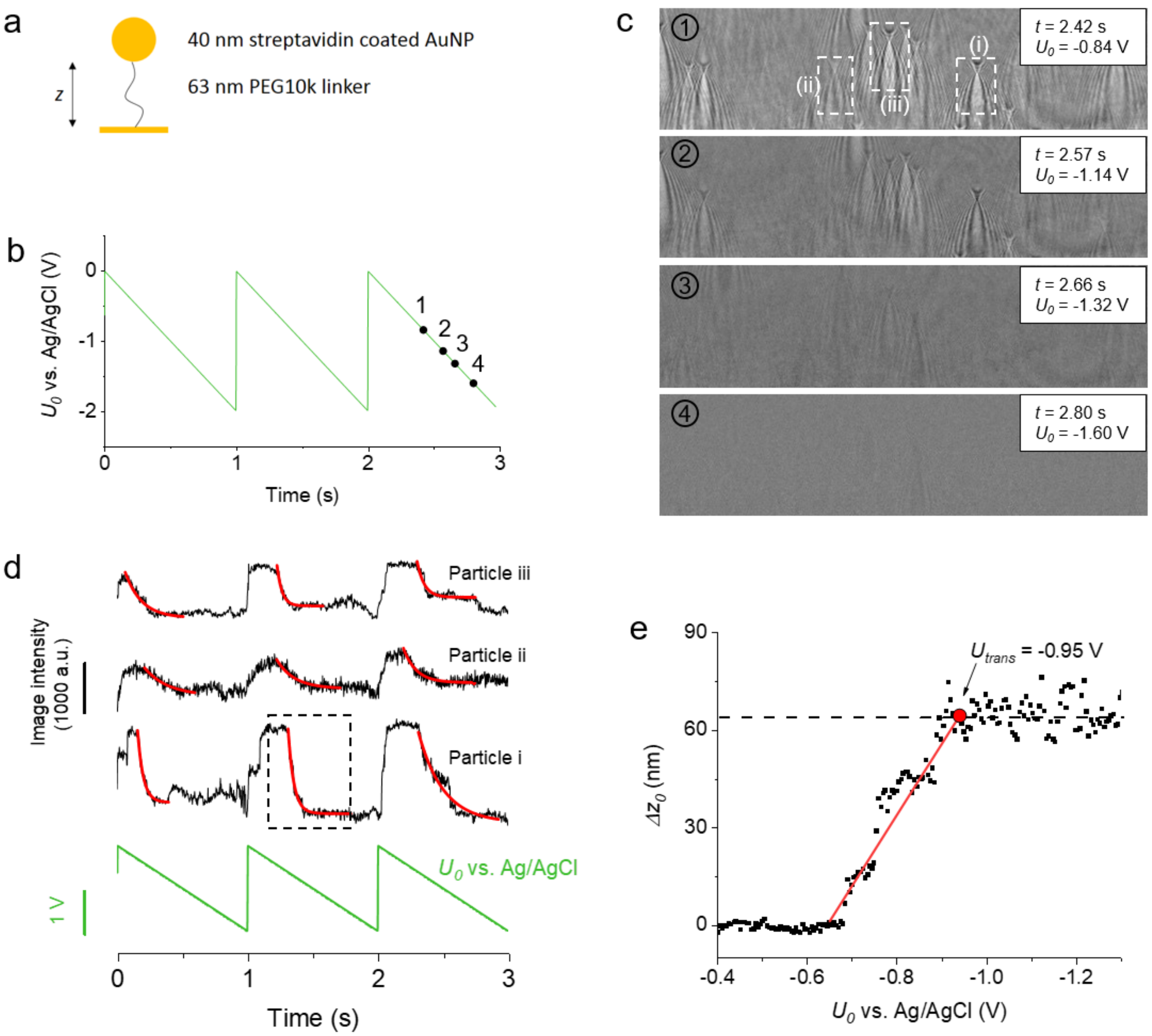

Supplementary Figure 1. Determining electric field with tethered gold nanoparticles (AuNPs). a, 40 nm streptavidin coated AuNPs are tethered to a gold surface via PEG linkers. Each particle is tethered by an SH-PEG10k-biotin linker (L_{PEG} = 63 nm) with a thiol and a biotin terminals. The thiol terminal binds to the gold surface and the biotin terminal binds to the particle via biotin-streptavidin interaction. **b**, Repeated linear sweep of the potential applied to the gold surface to drive the AuNPs. **c**, Snapshot images of the particles at different potentials marked in b, where the image intensity change reflects the particle-surface distance change. **d**, Image intensity changes of three particles marked in c (black curve) in response to the applied potential (green

curve), where the red curves are fitting of the data to an exponential decay function. **e**, Image intensity of particle i marked in d is converted to particle-surface distance (Δz_0), which is plotted as a function of potential, where the red line is the fitting of the linear regime, and the red dot marks the transition point between the linear and plateau regimes.

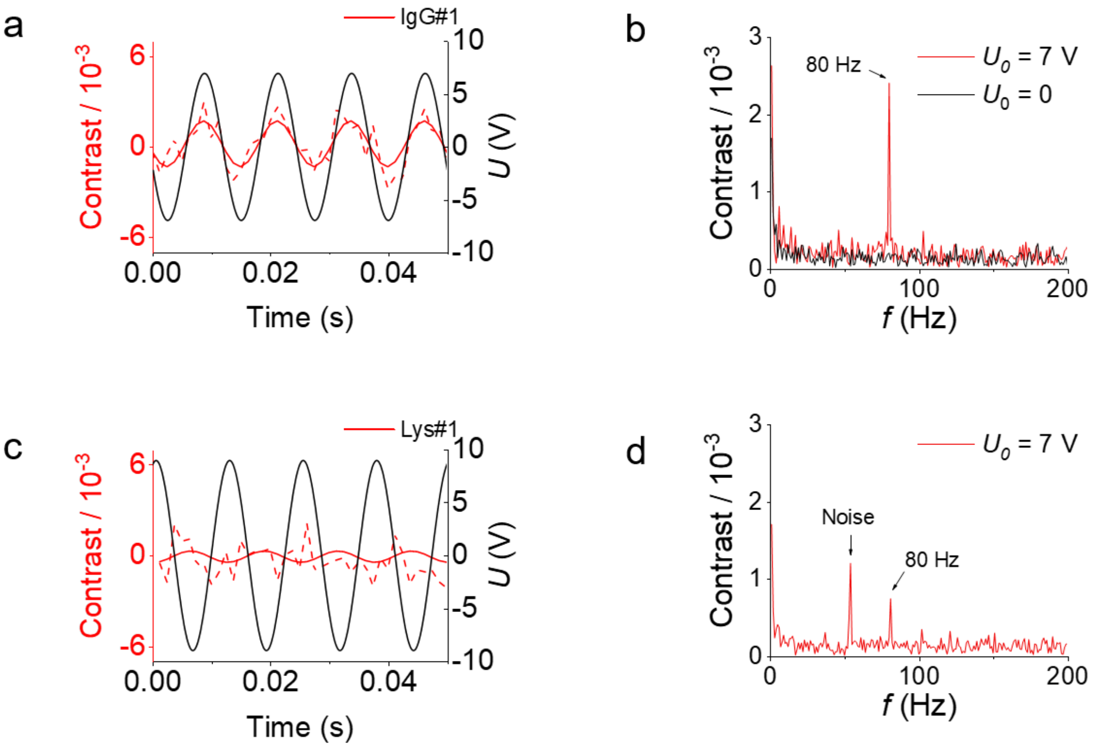

Supplementary Figure 2. Determine oscillation amplitude with FFT. **a**, Oscillation of an IgG molecule with potential (U_0), where the dashed red line shows the oscillation of the molecule and the solid red line is the oscillation after FFT filter at 80 Hz. The phase difference between the oscillation and potential is ~0°, indicating that IgG is negatively charged at pH = 7.4. **b**, FFT of the oscillation in a (***over one second)*** shows a peak at 80 Hz (red curve). No peak at 80 Hz is observed without applying electric field (black curve). **c**, Oscillation of a lysozyme (Lys) molecule with potential, where the dashed red line is the oscillation of the molecule and the solid red line is the oscillation after FFT filter at 80 Hz. The phase difference between the oscillation and potential is ~180°, indicating that lysozyme is positively charged at pH = 7.4. **d**, FFT of the oscillation of the lysozyme molecule in c over one second reveals a peak at 80 Hz. Note that the peak at 53 Hz is random noise. Buffer: 100× diluted PBS, pH = 7.4.

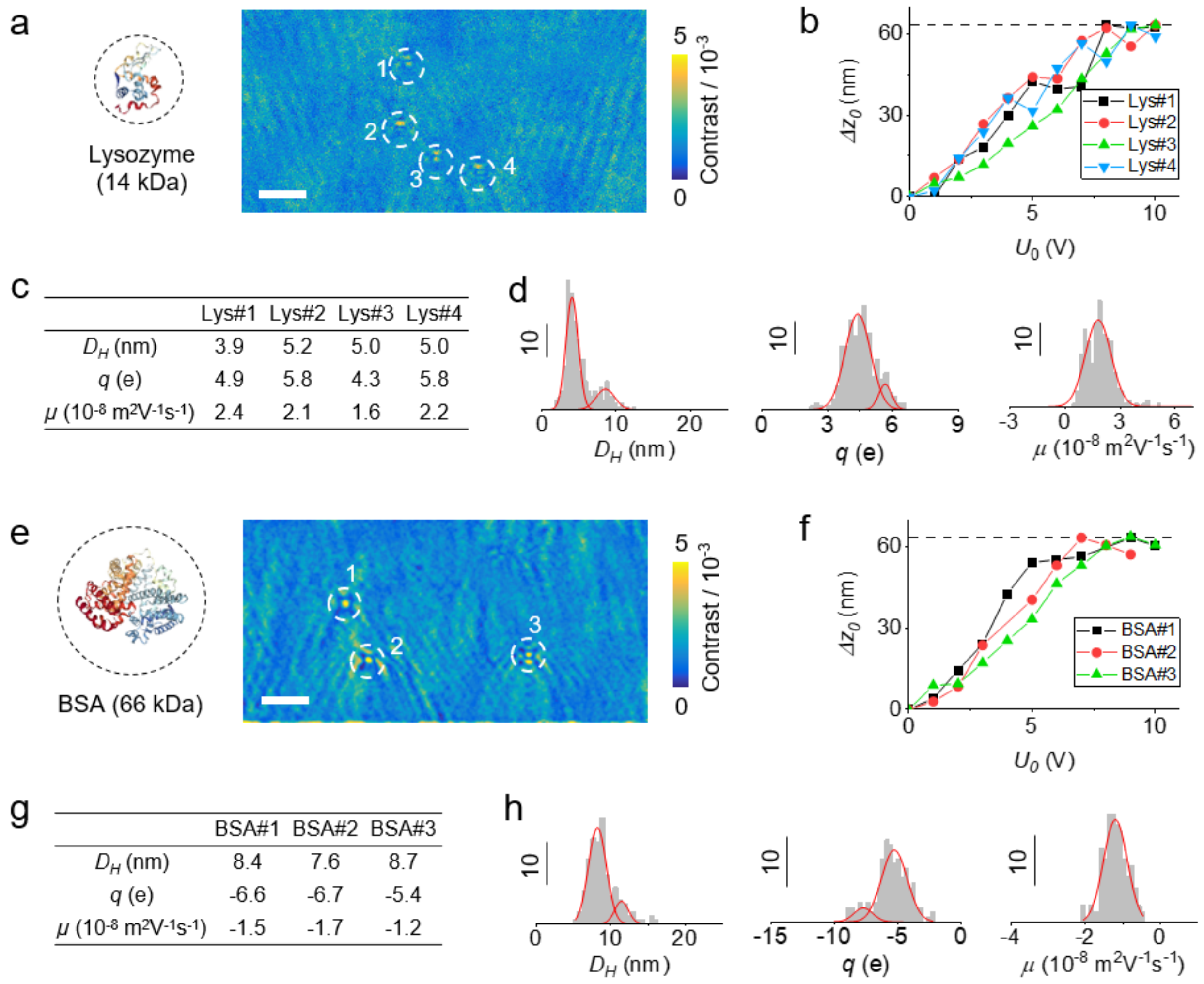

	Lys#1	Lys#2	Lys#3	Lys#4
D_H (nm)	3.9	5.2	5.0	5.0
q (e)	4.9	5.8	4.3	5.8
μ (10^{-8} $m^2V^{-1}s^{-1}$)	2.4	2.1	1.6	2.2

	BSA#1	BSA#2	BSA#3
D_H (nm)	8.4	7.6	8.7
q (e)	-6.6	-6.7	-5.4
μ (10^{-8} $m^2V^{-1}s^{-1}$)	-1.5	-1.7	-1.2

Supplementary Figure 3. Quantifying the size, charge and mobility of single lysozyme and BSA. a, FFT image of lysozyme molecules measured at U_0 = 9 V. **b**, Oscillation amplitude vs. U_0 plots of the lysozyme molecules marked in a, where the extracted D_H, q, and μ of the molecules are listed in **c**. **d**, Statistical analysis of 246 lysozyme molecules, where the red curves are Gaussian fittings to the histograms (see Supplementary Table 1). **e**, FFT image of BSA molecules obtained at potential of U_0 = 8 V. **f**, Oscillation amplitude vs. U_0 plots of the BSA molecules marked in e, where the extracted D_H, q, and μ are listed in **g**. **h**, Statistical analysis of 144 BSA molecules, where the red curves are Gaussian fittings to the histograms (see

Supplementary Table 1). In the diameter and charge histograms, small secondary peaks are observed in these proteins, which are due to dimers. Scale bars in a and e represent 3 μm.

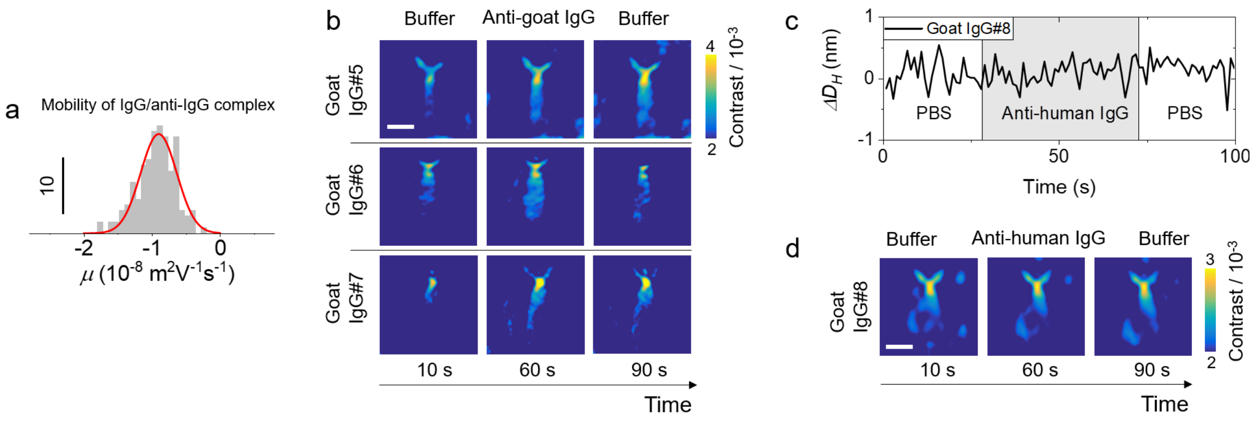

Supplementary Figure 4. Anti-IgG binding to IgG and control experiment. a, Mobility histogram of goat IgG molecules after incubation with 33 nM anti-goat IgG for 30 min. **b**, Snapshots of the IgG molecules in Fig. 2f during the binding experiment. **c**, A control experiment with anti-human IgG showing no detectable changes in the diameter of goat IgG. **d**, Snapshots of the IgG molecule in the control experiment. Scale bars: 3 µm.

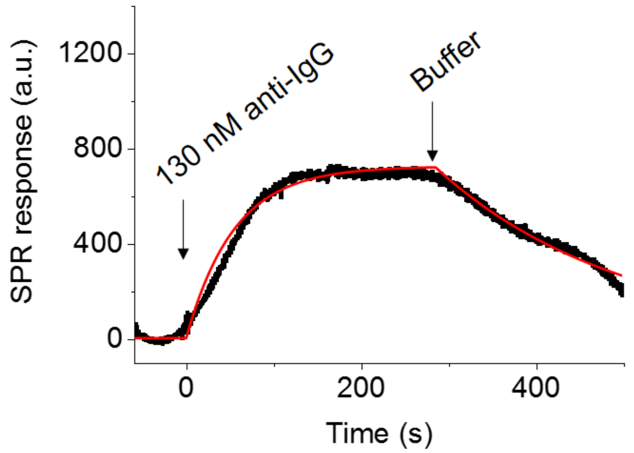

Supplementary Figure 5. Kinetics of anti-IgG binding to IgG measured with surface plasmon resonance (SPR), where the black line are experimental data and red line is fitting of the data to the first order kinetics, from which the association (k_a), dissociation (k_d) rate constants and the equilibrium constant (K_D) are determined to be 7.9×10^4 $M^{-1}s^{-1}$, 4.4×10^{-3} s^{-1}, and 55 nM, respectively.

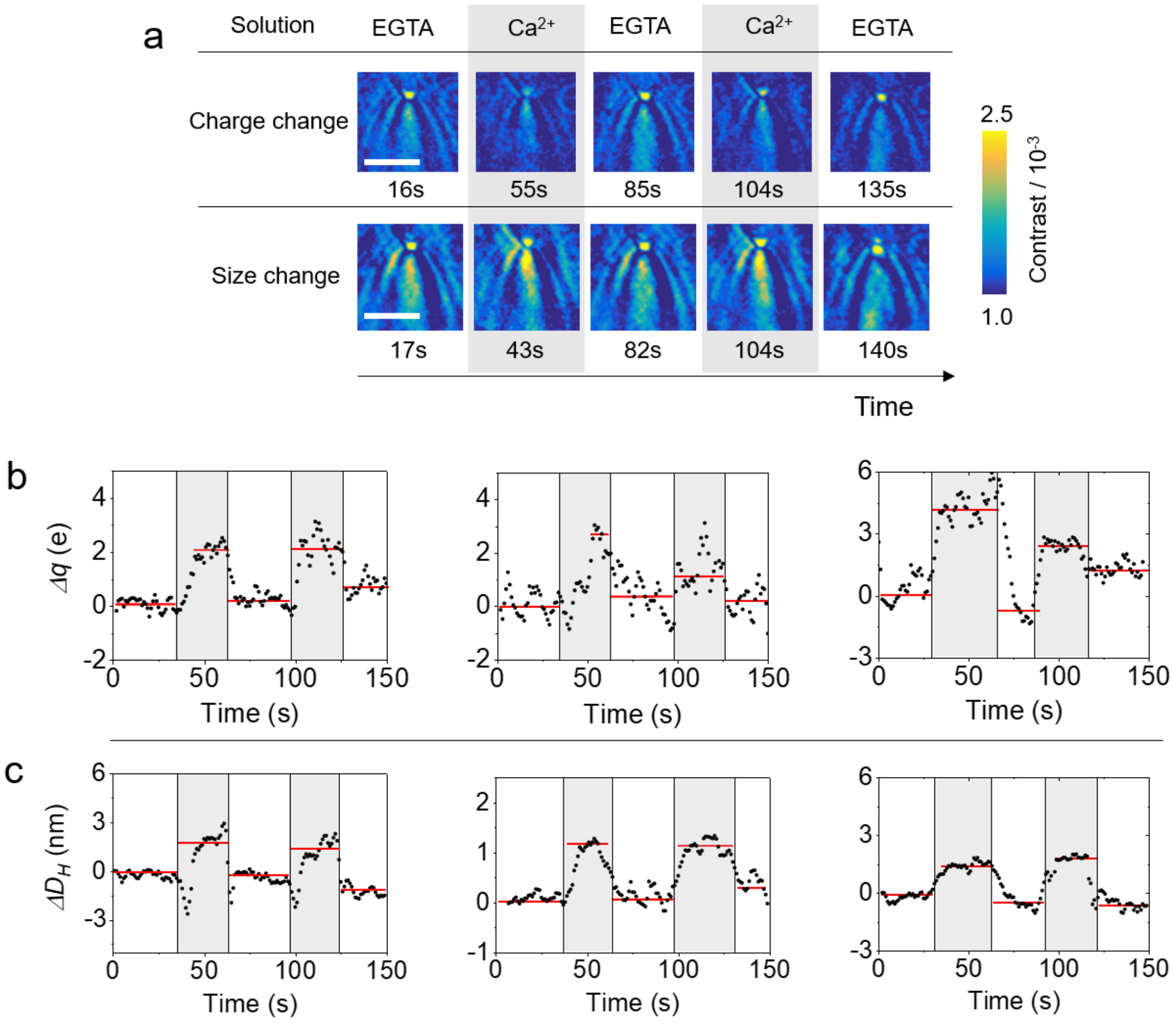

Supplementary Figure 6. Additional data on Ca^{2+} binding to CaM experiment. a, Snapshots of the CaM molecules in Figs. 3d and 3f captured during Ca^{2+} binding. Scale bars: 3 µm. **b**, Charge change and **c**, size change of CaM due to Ca^{2+} binding obtained by alternatively switching two solutions, 1 mM EGTA in 100× diluted PBS and 1 mM $CaCl_2$ in 100× diluted PBS at pH = 7.4. The black dots are raw data smoothened over 3 points, and the red lines are guide to the eye.

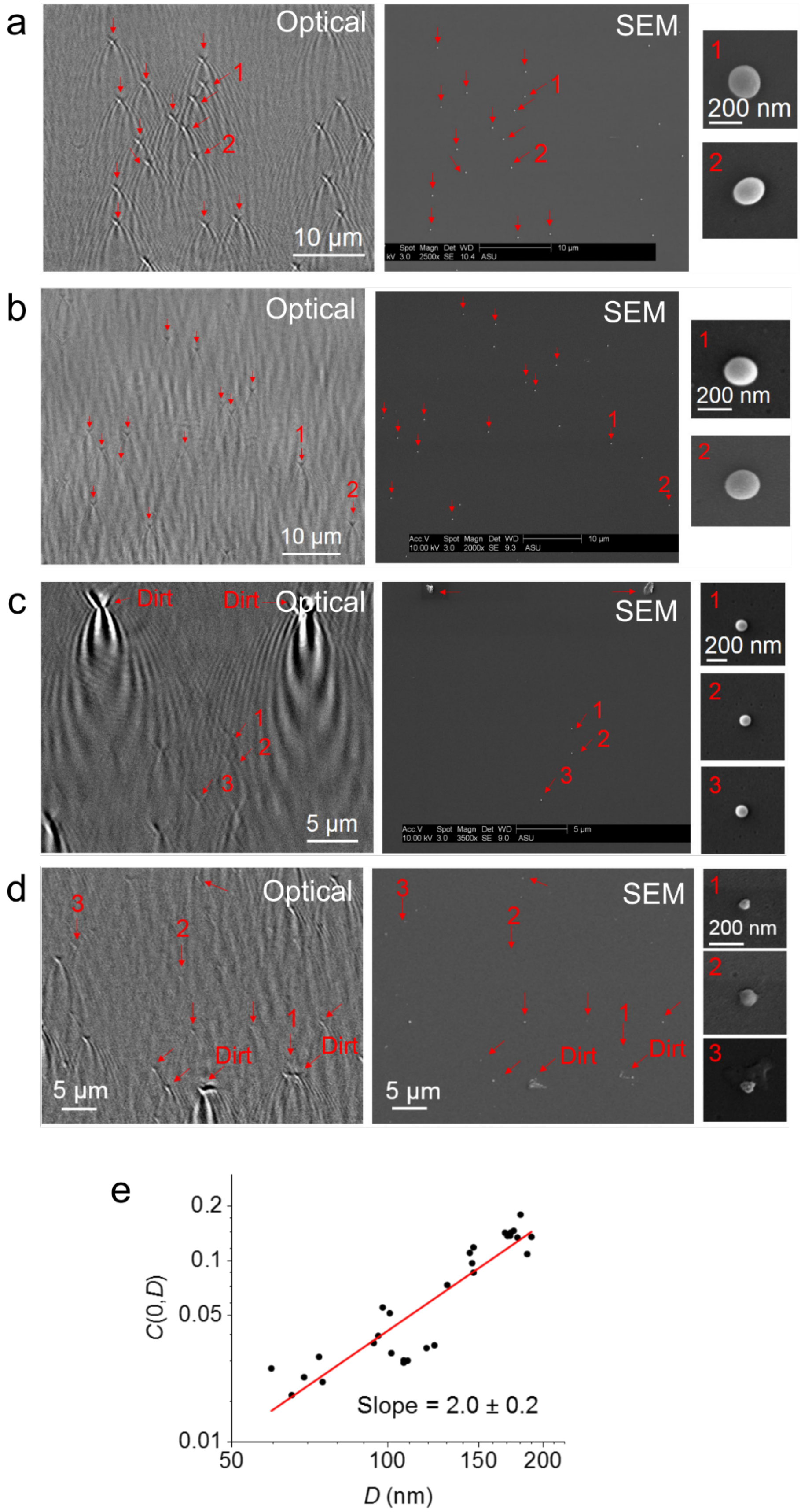

a
Optical
10 μm
SEM
1
2
200 nm
b
Optical
10 μm
SEM
c
Optical
Dirt
5 μm
SEM
3
d
Optical
Dirt
5 μm
SEM
e
0.2
0.1
0.05
0.01
50
100
150
200
C(0,D)
D (nm)
Slope = 2.0 ± 0.2

Supplementary Figure 7. Confirmation of single PS nanoparticles with SEM. **a-d**, Optical (left) and SEM (middle) images of nanoparticles of different sizes (190 nm, 145 nm, 99 nm, and 65 nm, respectively). The red arrows mark the same features in the optical image and the corresponding SEM image. Note that some large features (marked as dirt) in c and d serve as markers for SEM to locate the relatively small particles. The right panels show zoom-in of a few particles as indicated by numbers. **e**, Optical image contrast vs. diameter for single PS particles in logarithmic scale, showing a slope of 2.0 ± 0.2.

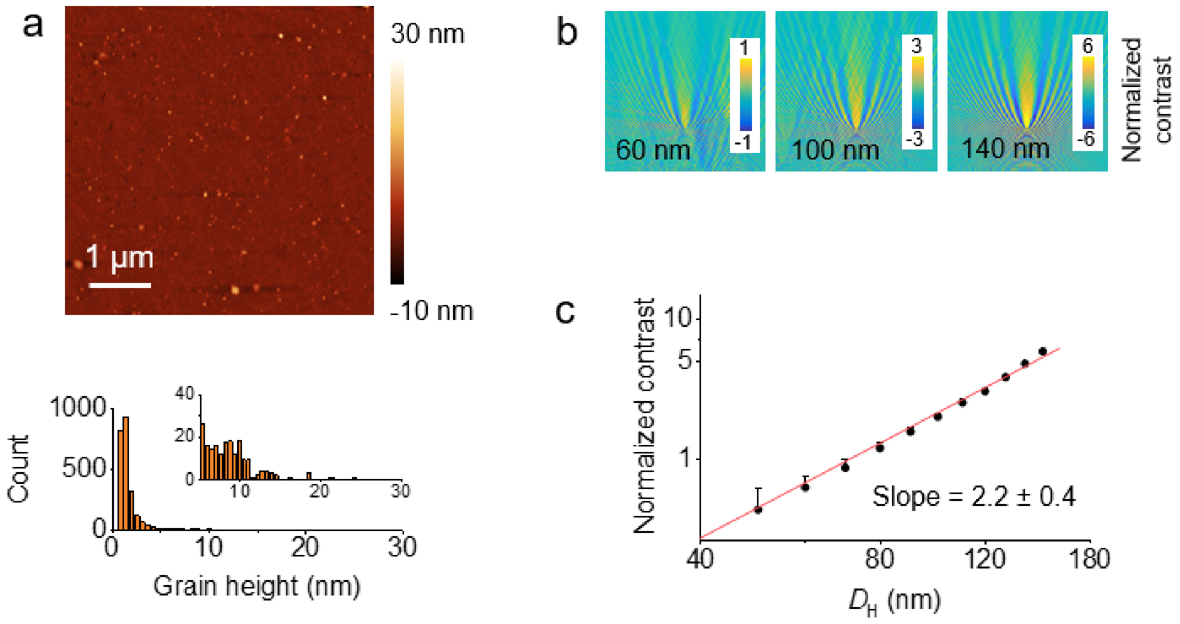

Supplementary Figure 8. Surface roughness effect. a, A typical AFM image of the ITO surface, showing grains with diameters ranging from 1 to 25 nm. The histogram shows size distribution of the grains. The inset shows a zoom-in of the histogram. **b**, Simulated images of polystyrene particles on the ITO surface. **c**, Image contrast of polystyrene particles vs. diameter in logarithmic scale, showing a slope of ~2.2. Note that each data point represents average over 10 particles.

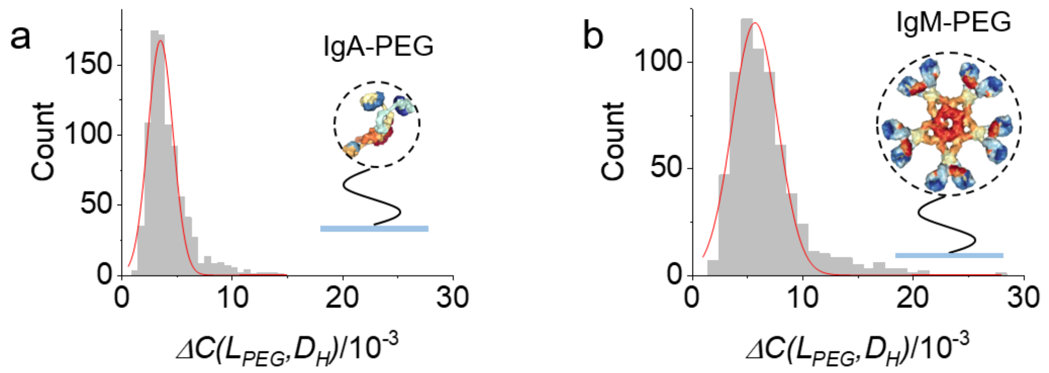

Supplementary Figure 9. Image contrast of IgA and IgM. a, Image contrast ($\Delta C(L_{PEG}, D_H)$) histogram of IgA-PEG obtained with 879 molecules. The contrast is $(3.8\pm1.9)\times10^{-3}$. **b**, Image contrast histogram of IgM-PEG obtained with 655 molecules. The contrast is $(6.2\pm3.2)\times10^{-3}$.

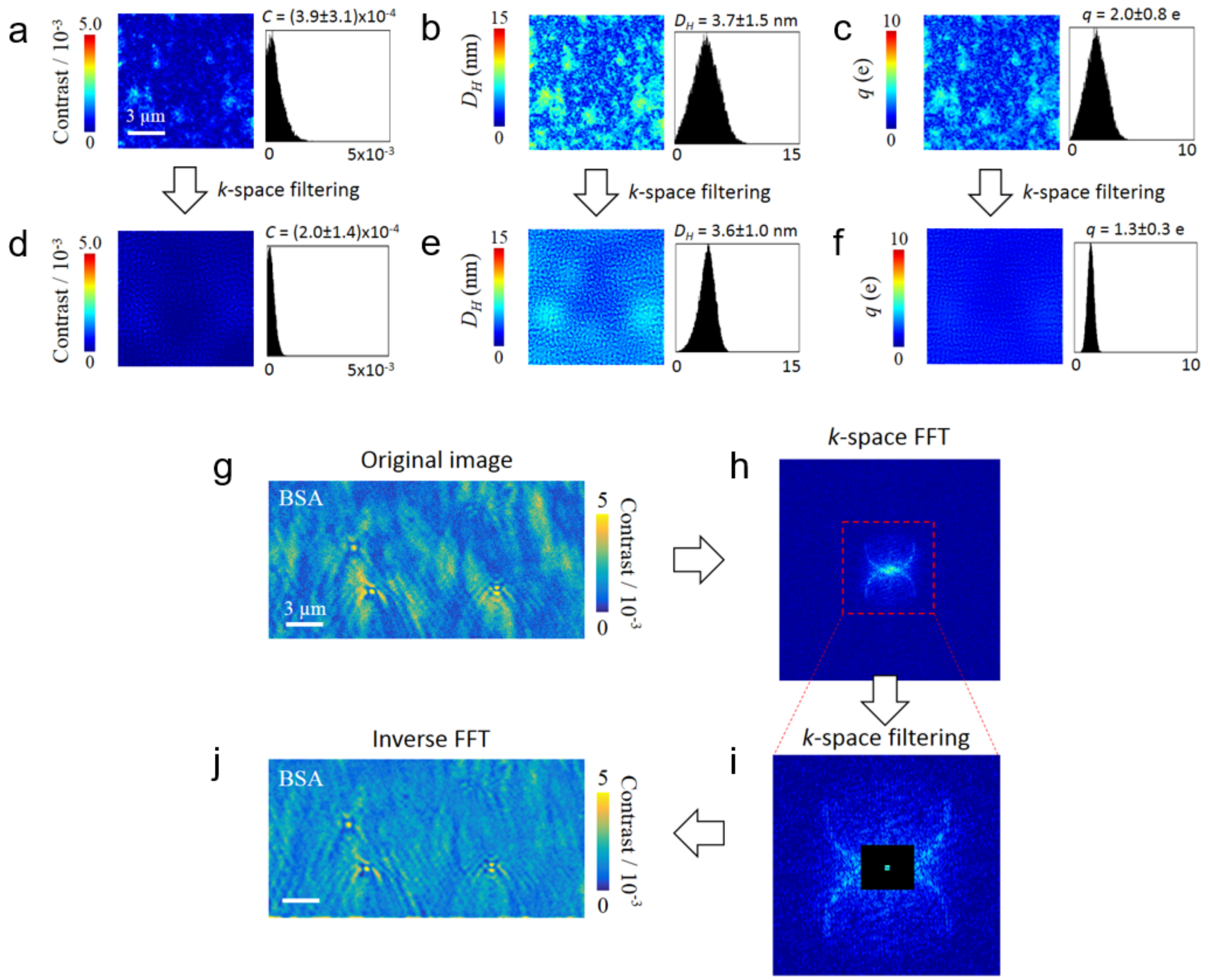

Supplementary Figure 10. Effect of ITO surface charging on image contrast, and measurement of diameter and charge of proteins. a, FFT image of a bare ITO surface by modulating the surface potential with amplitude, $U_0 = 10$ V and frequency, $f = 80$ Hz. **b**, The image contrast of each pixel in a is converted into diameter (D_H), showing a histogram with $D_H = 3.7 \pm 1.5$ nm. **c**, The image contrast of each pixel in a is converted into charge (q) with mobility of 1×10^{-8} $m^2V^{-1}s^{-1}$, and the charge histogram shows $q = 2.0 \pm 0.8$ e. **d-f**, The images in a-c are filtered in *k*-space with filter shown in i (black region) to reduce the background features. The image contrast, protein diameter and charge show histograms with $C = (2.0 \pm 1.4) \times 10^{-4}$, $D_H = 3.6 \pm 1.0$ nm, and $q = 1.3 \pm 0.3$ e, respectively. **g**, FFT image of single BSA molecules and background. **h**, *k*-space

FFT is performed with the FFT image in g, which shows two rings originated from interference. **i**, Zoom-in of the dashed region in h, where the black region is removed by the FFT filter. **j**, Inverse FFT of i shows BSA molecules with reduced the background spatial variation.

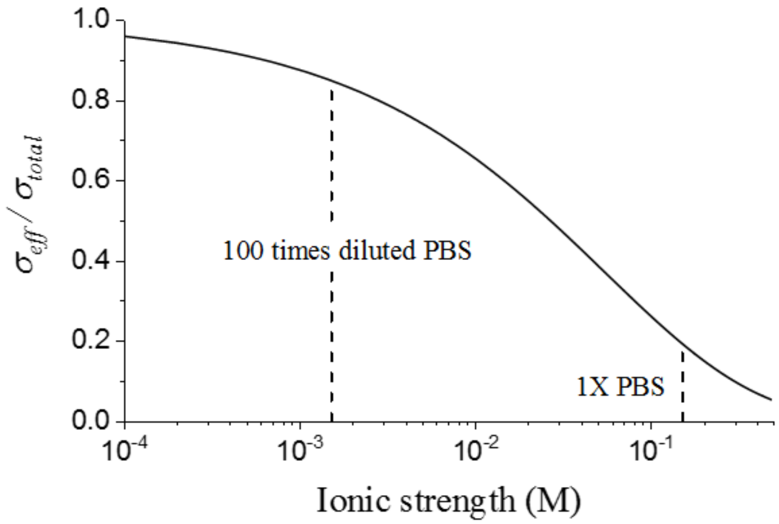

Supplementary Figure 11. Charge screening effect of PBS with different ionic strengths.

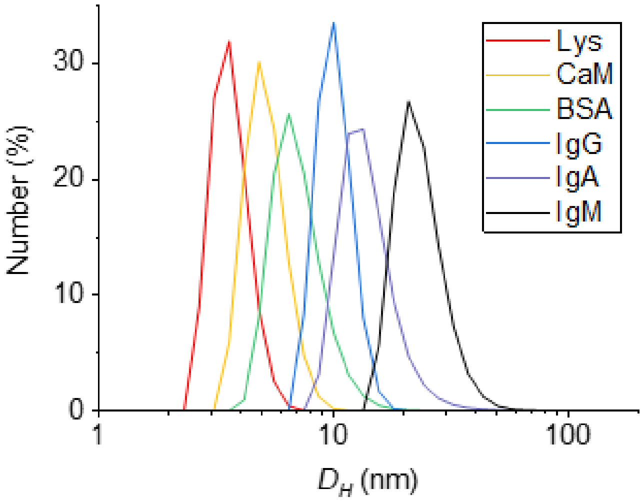

Supplementary Figure 12. Hydrodynamic diameter (*D_H*) of proteins measured with dynamic light scattering (DLS).

(a)

Error source	Measurement error	
	Size (D_H)	Charge (q)
Double layer charging	1 nm	0.3 e
Measuring the electric field	-	5%
PEG spring constant	-	7%
PEG length	3.3%	-
Measuring the oscillation plateau	3.3%	10%
Cumulative error	$0.05D_H + 1$ (nm)	$0.13\|q\| + 0.3$ (e)

(b)

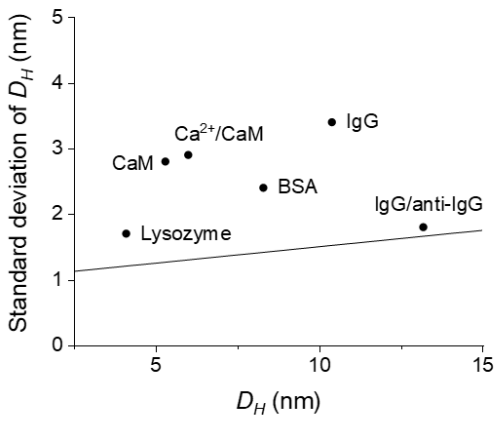

(c)

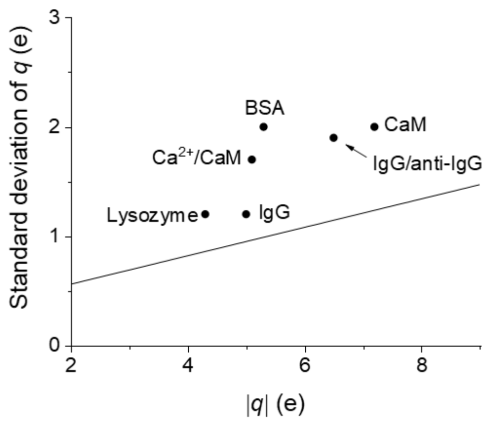

Supplementary Figure 13. Comparison between measurement error and experimental results. (a) A table showing factors that may induce measurement error and the value of the errors. All the values are based on calculations except the PEG spring constant is from literature.[1] The cumulative error is a function of size or charge. (b) and (c) Cumulative measurement error (solid line) and the standard deviation of size and charge obtained from experiments (dots).

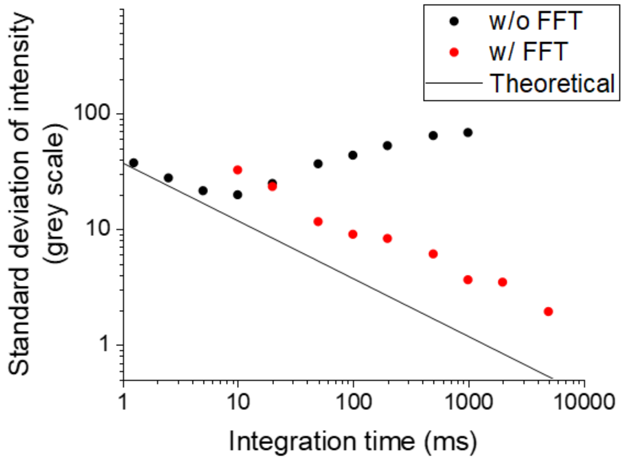

Supplementary Figure 14. Standard deviation of image intensity in the absence of particles at different integration times. An image sequence was recorded at 800 frames per second for 150 s on a bare ITO surface with or without electrical modulation (5V, 80 Hz). Then the images were integrated over different periods with or without the 80 Hz FFT filter. The new image sequences were differentiated, and the standard deviation of the image intensity in an area (10×10 pixels) of each image sequence was plotted vs. integration time. The red dots and the black dots represent with and without the FFT filter, respectively. The solid line is a prediction of the shot noise. The black dots (without FFT) deviates from the line for integration time longer than 10 ms, because mechanical drift becomes dominant. However, the red dots (with FFT) follows the line even after 5 s integration because the mechanical drift is filtered out. The red dots follow the trend of the line, which indicates shot noise is dominant. At 1 s integration time, FFT can reduce the noise by ~40 times. Note that the difference between the red dots and the line is due to other noises generated by the applied electrical field.

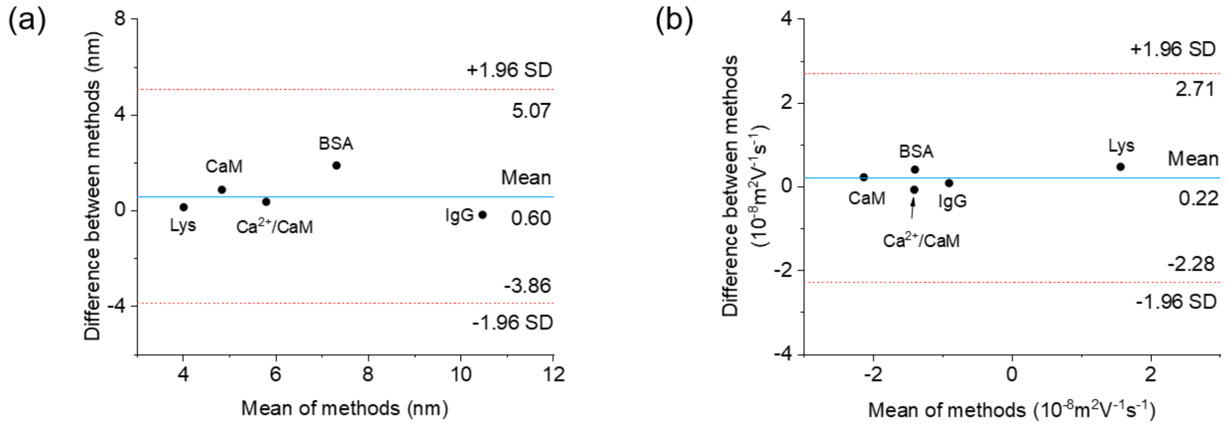

Supplementary Figure 15. Bland-Altman plots showing agreement between the hydrodynamic diameter (a) and mobility (b) measured by the method in this work and DLS.

Supplementary Table 1. Hydrodynamic diameter (D_H) of proteins reported in literature*

Protein	D_H (nm)	Method	Reference
**BSA**	6.6 to 8.6	DLS	2
	7	DLS	3
	7.3	DLS	4
**IgG**	10.58	Calculation	5
	10.9	DLS	6
	10.8 to 12.5	DLS	7
**Lysozyme**	3.7 to 3.9	DLS	8
	3.7	DLS	9
	4	Capillary electrophoresis	10
	4	NMR	11
**CaM**	4.96±0.18	NMR	11
	4.4	Calculation	12
	4.8	Gel permeation chromatography	12
	5.0±0.2	DLS	13
	4.44	SAXS	14
	4.1	SAXS	15
	4.1	SAXS	16
**Ca^{2+}/CaM**	4.90±0.08	NMR	11
	6.0±0.2	DLS	13
	4.3	SAXS	15

*DLS = dynamic light scattering, SAXS = small-angle X-ray scattering, NMR = nuclear magnetic resonance.

Supplementary Table 2. Mobility (μ) of proteins reported in literature*

Protein	μ ($\times 10^{-8}$ $m^2V^{-1}s^{-1}$)	Method	Reference
**BSA**	-1.4	ELS*	2
	-1.7	ELS	17
**IgG**	-0.8	Capillary electrophoresis	6
	-0.32	Capillary electrophoresis	18
**Lysozyme**	0.8	Electrophoresis and simulation	19
	0.15	ELS	20
	1.8	Capillary electrophoresis	10
	1.8	Capillary electrophoresis	21

*Note that mobility is sensitive to pH, ionic strength and ion species, and the mobilities measured in literature are not under the same experimental condition as in this work. Thus, small deviations are expected.

**ELS = electrophoretic light scattering.

Supplementary Table 3. Charges of proteins at pH = 7.4*

Protein	Charge at pH = 7.4	
	Net charge of amino acids	Estimation with zeta potential and size measured by Zetasizer (Malvern Panalytical)**
**BSA**	-14.0	-8.1
**IgG**	-0.6	-5.1
**Lysozyme**	8.2	2.4
**CaM**	-24.1	-6.6
**CaM with 4 Ca^{2+}**	-16.1	-4.1

*Note that the charge obtained from amino acids can be different from those measured by Zetasizer due to the binding of ions in solution.[22]

**Charge is estimated with Eq. 6 from the zeta potential and size of each protein.

Supplementary Table 4. Measured size (D_H), charge (q), and mobility (μ) of protein molecules and ligand-protein complexes.

	Molecules studied	D_H (SD*) nm	q (SD) e	μ (SD) 10^{-8} $m^2V^{-1}s^{-1}$
IgG	186	10.4 (3.4)	-5.0 (1.2)	-0.86 (0.39)
BSA	144	8.3 (2.4)	-5.3 (2.0)	-1.2 (0.75)
Lysozyme	246	4.1 (1.7)	4.3 (1.2)	1.8 (1.6)
CaM	150	5.3 (2.8)	-6.5 (1.9)	-2.0 (1.2)
Ca^{2+}/CaM	151	6.0 (2.9)	-5.1 (1.7)	-1.4 (1.1)
Anti-IgG/IgG	137	13.2 (1.8)	-7.2 (2.0)	-0.90 (0.62)

* SD = standard deviation.

Supplementary Note 1. Measurement error estimation.

Below are detailed descriptions for "Measurement error" in the Method section.

(a) PEG length. The PEG10k typically has a length variation of ~15% (example: https://www.sigmaaldrich.com/catalog/product/sial/81280?lang=en&region=US). Since the evanescent field decays exponentially from the surface, the most sensitive region is near the surface. Thus, the 15% variation does not introduce much difference in scattered light intensity. Here shows a calculation: The scattering intensity of an oscillating particle is given by, $\Delta I = I_0 - I_0 e^{-\frac{L_{PEG}}{d}}$, where I_0 is the intensity when the particle is close to the surface, *d* is the decay constant (about 100 nm), and L_{PEG} is the length of PEG. The average value of L_{PEG} is 63 nm and $\Delta I = 0.47 I_0$. When L_{PEG} is 15% longer, $\Delta I = 0.52 I_0$. Thus, the difference in ΔI is ~10%, leading to $\sqrt{10}\% = 3.3\%$ difference in size (because intensity is proportional to the square of diameter, as shown in Fig. 4a).

(b) PEG spring constant. The variation in spring constant is ~7% measured by AFM force spectroscopy.[1] The spring constant could affect the accuracy in charge measurement but does not affect size measurement, because the size is determined by the image intensity when the PEG is fully stretched by sufficiently high potential.

(c) Determining the plateau of oscillation. The variation in *z* at the plateau is ~10 nm (e.g., Fig. 2b), which leads to ~10% intensity change (or 3.3% size change) according to $\Delta I = I_0 - I_0 e^{-\frac{L_{PEG}}{d}}$. This variation also introduces 10% uncertainty in charge.

(d) Measuring the applied electric field. The charge of the gold nanoparticles in Supplementary Fig. 1 is measured by DLS and the value is -42.5±2.3 e, which causes 5.4% uncertainty in the measured field. The transition potential (U_{trans}) determined from each cycle is slightly different

even for the same particle (Supplementary Fig. 1d). This is due to charge fluctuation, which is intrinsic for charged particles.[23, 24] We have determined U_{trans} from 21 cycles using 7 nanoparticles shown in Supplementary Fig. 1c, which is –(0.93±0.08) V. Thus, the uncertainty is 8.7%. Since the variation in U_{trans} arises from the variation in the charge of each nanoparticle, this uncertainty should not be included in error propagation.

Supplementary Note 2. MATLAB code for image FFT.

```
% FFT images
% This code was originally written by Dr. Xiaonan Shan and modified by G.M.

clear all; close all; warning off;
directory_name = uigetdir;
filelist = dir([directory_name,'\','*.tif']);   % Select folder
[N,M] = size(imread([directory_name,'\', filelist(1).name]));
FrameRate = 800;    % Frame rate of the images (images per second)

LoadFrame=800;  % The number of images used to perform FFT
Images = zeros(N,M,LoadFrame,'single');

StartFM = 1;
firstImg = single(imread([directory_name,'\', filelist(StartFM).name]));

xlsfiles={filelist.name};
xlsfiles=sort_nat(xlsfiles);      % Sort image names in natural order. Douglas Schwarz (2020).
sort_nat: Natural Order Sort (https://www.mathworks.com/matlabcentral/fileexchange/10959-
sort_nat-natural-order-sort), MATLAB Central File Exchange. Retrieved August 21, 2020.

a = waitbar(0);
for i = StartFM:StartFM+LoadFrame-1
   waitbar((i-StartFM+1)/(LoadFrame),a,'Loading images...');
   Images(:,:,i-StartFM+1) = single(imread([directory_name,'\', char(xlsfiles(i))]))-firstImg;
end
close(a);

TempFFT = fft(Images,[],3);
Amplitude = abs(TempFFT)/LoadFrame*2;   % The value of each pixel represents oscillation
amplitude (unit: gray scale)

WholeSurfFFT = fft(squeeze(mean(mean(Images))));
WholeSurfFFTAmp = abs(WholeSurfFFT);
a = 1:LoadFrame;
figure; % Plot frequency spectrum.
plot (a./LoadFrame.*FrameRate,WholeSurfFFTAmp(a));
title('Amplitude vs. Frequency');
xlabel('Frequency (Hz)')
ylabel('Amplitude (arb. unit)')

figure; % Show FFT images at each frequency
for j =1:LoadFrame
imagesc(filter2(ones(5,5),Amplitude(:,:,j)),[1 2000]);
title([num2str((j-1)/LoadFrame*FrameRate),' Hz']);
```

```
pause();
end

imwrite(uint16(Amplitude(:,:,81)),'FFT_image_at_80Hz.tiff');
```

References

1. Maaloum, M. & Courvoisier, A. Elasticity of Single Polymer Chains. *Macromolecules* **32**, 4989-4992 (1999).
2. Jachimska, B., Wasilewska, M. & Adamczyk, Z. Characterization of Globular Protein Solutions by Dynamic Light Scattering, Electrophoretic Mobility, and Viscosity Measurements. *Langmuir* **24**, 6866-6872 (2008).
3. Yu, S. et al. Albumin-coated SPIONs: An experimental and theoretical evaluation of protein conformation, binding affinity and competition with serum proteins. *Nanoscale* **8**, 14393-14405 (2016).
4. Li, Y., Yang, G. & Mei, Z. Spectroscopic and dynamic light scattering studies of the interaction between pterodontic acid and bovine serum albumin. *Acta Pharmaceutica Sinica B* **2**, 53-59 (2012).
5. Armstrong, J.K., Wenby, R.B., Meiselman, H.J. & Fisher, T.C. The hydrodynamic radii of macromolecules and their effect on red blood cell aggregation. *Biophysical journal* **87**, 4259-4270 (2004).
6. Bermudez, O. & Forciniti, D. Aggregation and denaturation of antibodies: a capillary electrophoresis, dynamic light scattering, and aqueous two-phase partitioning study. *Journal of Chromatography B* **807**, 17-24 (2004).
7. Sukumar, M., Doyle, B.L., Combs, J.L. & Pekar, A.H. Opalescent appearance of an IgG1 antibody at high concentrations and its relationship to noncovalent association. *Pharmaceutical research* **21**, 1087-1093 (2004).
8. Parmar, A.S. & Muschol, M. Hydration and hydrodynamic interactions of lysozyme: effects of chaotropic versus kosmotropic ions. *Biophysical journal* **97**, 590-598 (2009).
9. Grigsby, J., Blanch, H. & Prausnitz, J. Diffusivities of lysozyme in aqueous MgCl2 solutions from dynamic light-scattering data: effect of protein and salt concentrations. *The Journal of Physical Chemistry B* **104**, 3645-3650 (2000).
10. Sharma, U. & Carbeck, J.D. Hydrodynamic radius ladders of proteins. *Electrophoresis* **26**, 2086-2091 (2005).
11. Weljie, A.M., Yamniuk, A.P., Yoshino, H., Izumi, Y. & Vogel, H.J. Protein conformational changes studied by diffusion NMR spectroscopy: Application to helix-loop-helix calcium binding proteins. *Protein Science* **12**, 228-236 (2003).
12. Sorensen, B.R. & Shea, M.A. Calcium binding decreases the stokes radius of calmodulin and mutants R74A, R90A, and R90G. *Biophysical Journal* **71**, 3407-3420 (1996).
13. Papish, A.L., Tari, L.W. & Vogel, H.J. Dynamic Light Scattering Study of Calmodulin–Target Peptide Complexes. *Biophysical Journal* **83**, 1455-1464 (2002).
14. Majava, V. et al. Interaction between the C-terminal region of human myelin basic protein and calmodulin: analysis of complex formation and solution structure. *BMC Structural Biology* **8**, 10 (2008).
15. Seaton, B., Head, J., Engelman, D. & Richards, F. Calcium-induced increase in the radius of gyration and maximum dimension of calmodulin measured by small-angle X-ray scattering. *Biochemistry* **24**, 6740-6743 (1985).
16. Majava, V. & Kursula, P. Domain Swapping and Different Oligomeric States for the Complex Between Calmodulin and the Calmodulin-Binding Domain of Calcineurin A. *PLOS ONE* **4**, e5402 (2009).

17. Takeda, K. et al. Size and mobility of sodium dodecyl sulfate—bovine serum albumin complex as studied by dynamic light scattering and electrophoretic light scattering. *Journal of Colloid and Interface Science* **154**, 385-392 (1992).
18. Martin, N. et al. Prevention of thermally induced aggregation of IgG antibodies by noncovalent interaction with poly (acrylate) derivatives. *Biomacromolecules* **15**, 2952-2962 (2014).
19. Yamaguchi, A. & Kobayashi, M. Quantitative evaluation of shift of slipping plane and counterion binding to lysozyme by electrophoresis method. *Colloid and Polymer Science* **294**, 1019-1026 (2016).
20. Cugia, F., Monduzzi, M., Ninham, B.W. & Salis, A. Interplay of ion specificity, pH and buffers: insights from electrophoretic mobility and pH measurements of lysozyme solutions. *RSC Advances* **3**, 5882-5888 (2013).
21. Szymański, J.d. et al. Net charge and electrophoretic mobility of lysozyme charge ladders in solutions of nonionic surfactant. *The Journal of Physical Chemistry B* **111**, 5503-5510 (2007).
22. Yang, D., Kroe-Barrett, R., Singh, S. & Laue, T. IgG Charge. *Preprints* (2018).
23. Shan, X. et al. Detection of charges and molecules with self-assembled nano-oscillators. *Nano letters* **14**, 4151-4157 (2014).
24. Ma, G., Shan, X., Wang, S. & Tao, N. Quantifying Ligand–Protein Binding Kinetics with Self-Assembled Nano-oscillators. *Analytical Chemistry* **91**, 14149-14156 (2019).
