# Supplementary material for: Optical imaging of single-protein size, charge, mobility, and binding
Source: Nat Commun. 2020 Sep 21;11:4768. doi: 10.1038/s41467-020-18547-w (PMC7505846; doi:10.1038/s41467-020-18547-w)
Supplement: Supplementary file 3 — Description of Additional Supplementary Files [file 41467_2020_18547_MOESM3_ESM.docx]

**Description of Additional Supplementary Files**

**File Name: Supplementary Video 1**

**Description:** Anti-IgG binding to an IgG molecule and resulted size change over time. An IgG molecule is driven into oscillation in the plateau regime (U0 = 9 V, and f = 80 Hz). 100× diluted PBS buffer is flown over the IgG coated surface for the first 25 seconds, then 130 nM anti-IgG is introduced to the solution. Image size: 4 × 4 µm2, and image contrast scale: 2-4×10-3.

**File Name: Supplementary Video 2**

**Description:** Ca2+ binding to CaM and resulted charge change over time. A CaM molecule is driven into oscillation with U0 = 4 V, and f = 80 Hz. 100× diluted PBS solutions with EGTA and Ca2+ were alternatively flown over the surface. Image size: 6.5 × 6.5 µm2; Image contrast scale: 1-3×10-3.
